# Data on European seabass fed with methionine-enriched diets obtained through label free shotgun proteomics

**DOI:** 10.1016/j.dib.2020.106675

**Published:** 2020-12-19

**Authors:** Ana Paula Farinha, Denise Schrama, Tomé Silva, Luís E.C. Conceição, Rita Colen, Sofia Engrola, Pedro Rodrigues, Marco Cerqueira

**Affiliations:** a Centre of Marine Sciences (CCMAR), Universidade do Algarve, Campus de Gambelas, 8005-139 Faro, Portugal; b Universidade do Algarve, Campus de Gambelas, 8005-139 Faro, Portugal; c SPAROS Lda., Área Empresarial de Marim, Lote C, 8700-221 Olhão, Portugal

**Keywords:** *Dicentrarchus labrax*, Nutritional proteomics, Methionine, Farmed fish, Liver proteome, Metabolism

## Abstract

This data article is associated with the research article “Evaluating the impact of methionine-enriched diets in the liver of European seabass through label-free shotgun proteomics”. Here it is described the data obtained from proteomic analysis of 36 European seabass juveniles (3 fish x 3 replicate tanks) after 18 days of feeding with experimental diets containing four inclusion levels of methionine (Met): 0.77%, 1%, 1.36% and 1.66% Met (w/w). We analysed this dataset and compared it with that obtained during the long-term feeding period i.e., 85 days. Fish liver proteins were digested with trypsin and purified peptides were analysed by LC-MS/MS. Proteins were identified with at least two peptides at 0.1% Decoy false discovery rate (FDR). In this dataset, we present the analysis of the differential abundant proteins (DAP) with significant differences across treatments after 18 days of feeding (One-Way ANOVA, *p* < 0.05). Treatment's comparisons were also performed between the 18- and 85-days feeding trials through Two-Way ANOVA (*p* < 0.05). MS/MS raw data are available via ProteomeXChange with identifiers PXD019610 and 10.6019/PXD019610 (18-days dataset); and PXD019622 and 10.6019/PXD019622 (85-days dataset). This dataset corresponds to fish sampled after 18-days of experimental trial and is made available to support the study conducted in the afore-mentioned article, by performing the analysis during a short-term period of feeding. The data presented may be further used in other nutritional studies e.g., addressing hepatic changes mediated by Met.

## Specifications Table

**Table:** 

Subject	Biology
Specific subject area	Fish proteomics, Label-free quantitative mass spectrometry
Type of data	Figure Table
How data were acquired	Mass spectrometry, by using reverse phase nano liquid chromatography (Ultimate 3000 system) coupled online to a Q-Exactive Hybrid Quadrupole-Orbitrap mass spectrometer (Thermo Scientific, Bremen, Germany) in data-dependent acquisition mode. Scaffold software (version Scaffold_4.8.9, Proteome Software Inc., Portland, OR) was used to validate MS/MS based peptide and protein identifications, and perform quantitative analysis based on extracted ion chromatograms and for the TOP3 resulting most intense ion peak areas.
Data format	Raw data Analysed data through uni- and multi-variate statistical analyses.
Parameters for data collection	Liver protein samples were obtained from fish fed with the following dietary methionine (Met) inclusion levels: 0.77% Met (w/w), 1% Met (w/w), 1.36% Met (w/w) and 1.66% Met (w/w); diets M0.65, M0.85, M1.25 and M1.5, correspondingly.
Description of data collection	The four experimental diets were tested in triplicate and 3 fish/tank were randomly sampled for proteomic analysis, in a total of 9 fish/treatment. Protein samples were analyzed by label free shotgun proteomics (LC-MS/MS).
Data source location	European seabass juveniles were reared at Ramalhete Field Station (CCMAR, Universidade do Algarve, Faro) - Portugal, between May and August of 2018.
Data accessibility	Data are with this article and the MS/MS raw and msf files have been deposited to the ProteomeXChange Consortium via the PRIDE repository and are publicly available on http://www.ebi.ac.uk/pride/archive/projects/PXD019610 Data identifiers: PXD019610 and 10.6019/PXD019610
Related research article	A.P. Farinha, D. Schrama, T. Silva, L.E.C. Conceição, R. Colen, S. Engrola, P. Rodrigues, M. Cerqueira. Evaluating the impact of methionine-enriched diets in the liver of European seabass through label-free shotgun proteomics. Journal of Proteomics. In Press. https://doi.org/10.1016/j.jprot.2020.104047

## Value of the Data

• These label free shotgun proteomics data are important as it represents the first comprehensive dataset on the liver proteome of European seabass in response to dietary methionine, being compared between a short-term and long-term period of feeding i.e., 18 and 85 days of an experimental trial.
• Both private and public aquaculture sectors can benefit from these data, as it refers to farmed fish with economic relevance, especially in the Mediterranean region. The data provides also relevant information about regulatory mechanisms involved in cell/organism response to methionine, that can be further extended to other teleost fish, besides vertebrate organisms.
• The current proteomics data offer complementary information to classical fish performance indicators, that may be useful in the refinement of amino acid requirements, in this case methionine, assessing fish hepatic proteome changes over a short-term and long-term period of feeding.
• As methionine is an indispensable amino acid with an essential role in both fish growth and immune condition/response, these data can help to define future protein indicators/biomarkers of growth performance and/or of immune condition.

## 1 Data Description

**Fig. 1:**
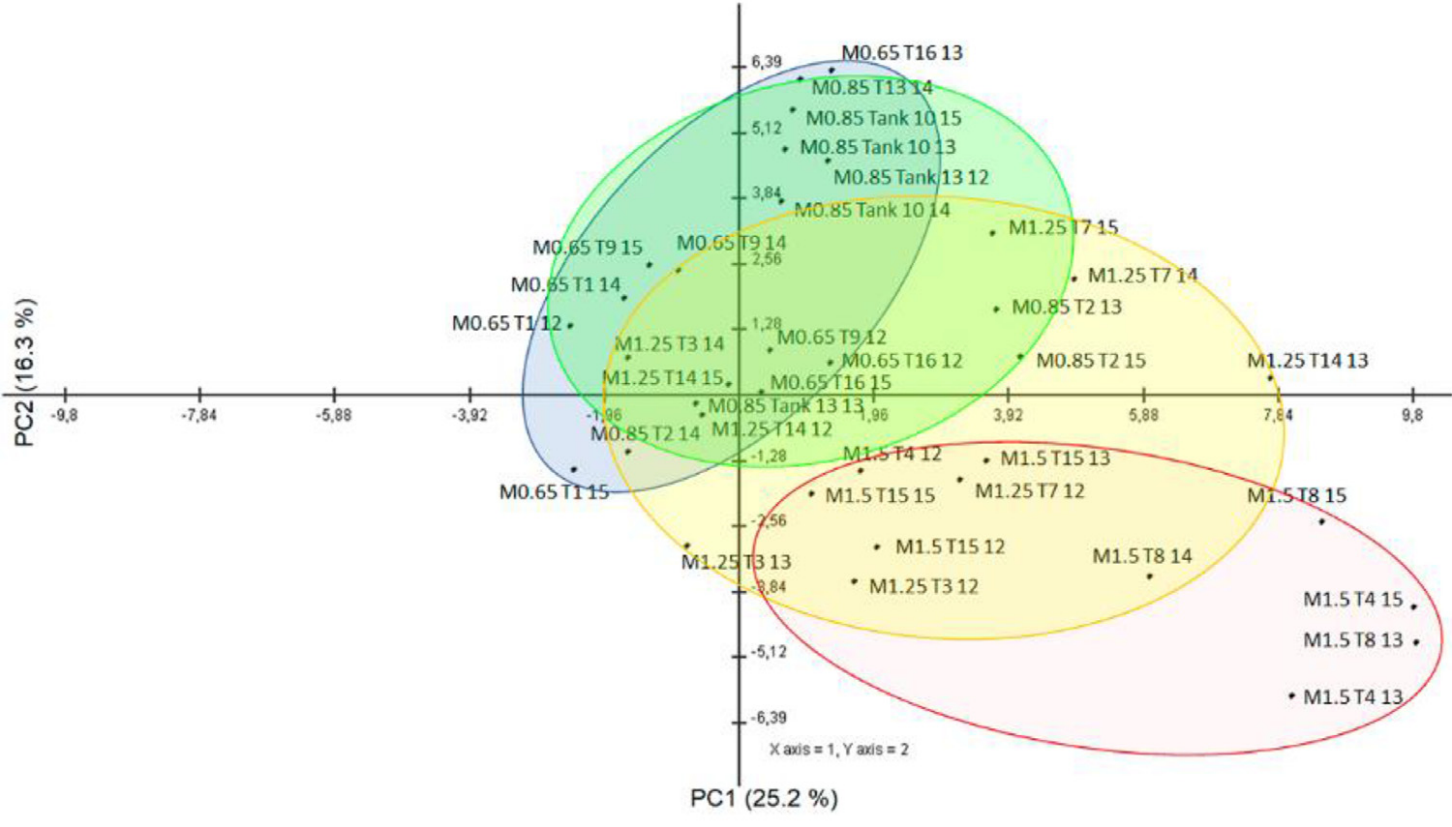
Principal component analysis of DAP with most significant differences (One-Way ANOVA, *p* < 0.01) after 18 days of feeding European seabass juveniles with experimental diets. Fish were fed with the following Met-inclusion levels: 0.77%, 1%, 1.36% and 1.66% Met (w/w); M0.65, M0.85, M1.25 and M1.5, correspondingly.

**Fig. 2:**
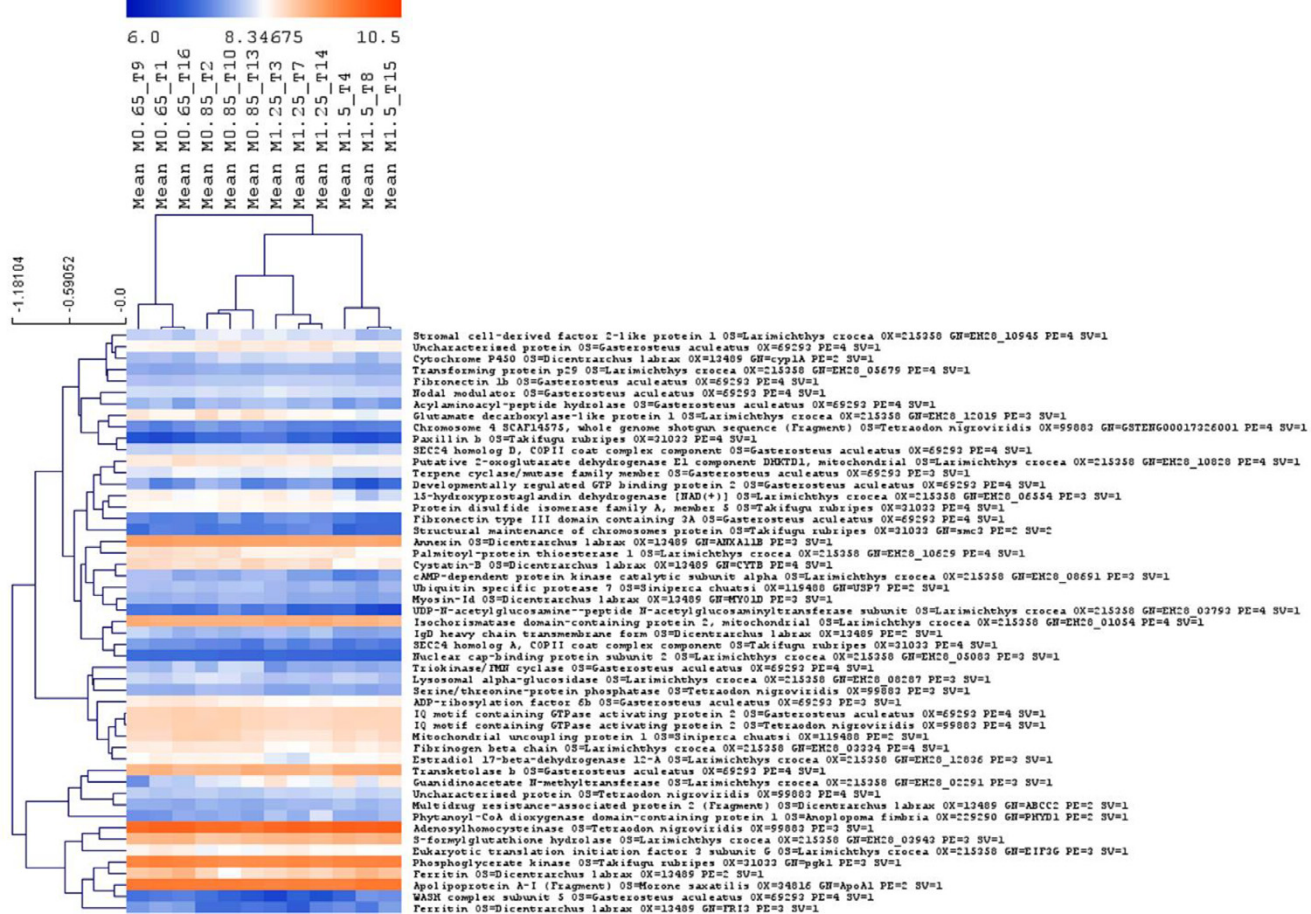
Hierarchical clustering analysis (HCL) after 18 days of feeding fish fed with Met-enriched diets. Clustering of fish samples and proteins with significant changes between treatments (One-Way ANOVA, *p* < 0.05). The mean values of protein abundance were obtained from 3 liver samples/tank and triplicate tanks per treatment are shown.

**Fig. 3:**
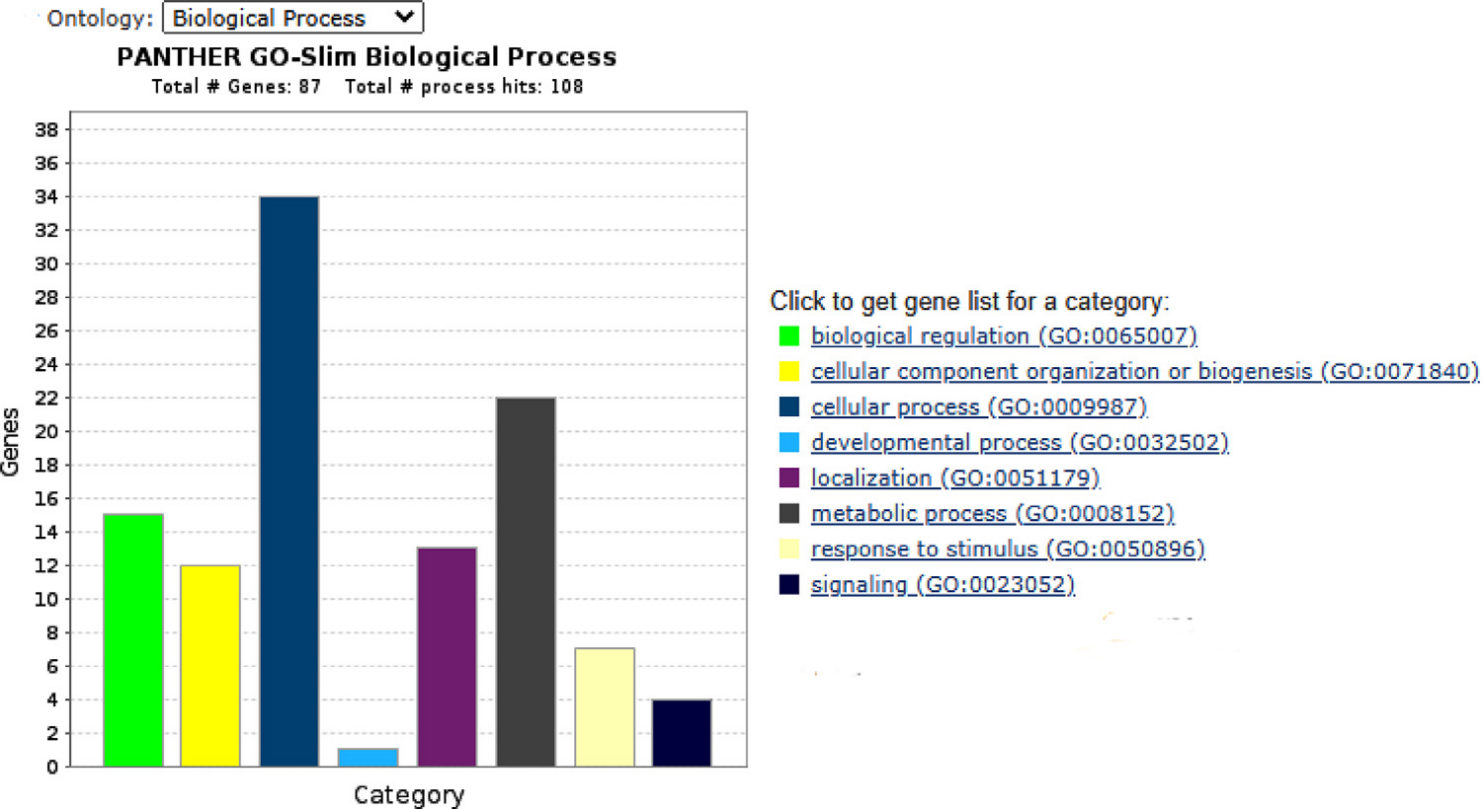
Gene Ontology (GO) classification of proteins with significant differences (One-way ANOVA, *p* < 0.05) between fish fed with distinct Met-supplemented diets, according to Biological Process.

This proteomic dataset was obtained from European seabass juveniles fed with different levels of Met-supplementation during a short-term period of feeding i.e., 18 days. The results of the proteomics data obtained at the end of the trial (after 85 days of feeding) are discussed in the associated research article entitled “Evaluating the impact of methionine-enriched diets in the liver of European seabass through label-free shotgun proteomics” [1]. The present proteomics data aims to offer additional information of the fish liver response to dietary Met-at an earlier time point i.e., after 18 days of feeding. The TOP3 precursors’ intensity was used for quantitation of protein relative abundance between samples. A Principal Component Analysis (PCA) of proteins with most significant differences between fish fed with the different experimental diets (ANOVA, *p* < 0.01) was performed to discriminate against the four Met-treatment groups (Fig. 1). Additional analysis through One-way ANOVA (*p* < 0.05) combined with multivariate hierarchical clustering (HCL) based on Pearson's correlation, were conducted to infer about samples’ and protein's relationship based on quantitative values of relative protein abundance (Fig. 2). A Gene Ontology (GO) classification of the liver proteins showing significant differences across treatments (One-way ANOVA, *p* < 0.05) was established according to the PANTHER 14.1 classification system [2] to disclose most relevant Biological Processes (BP) implicated in the hepatic response to dietary Met (Fig. 3).

**Fig. 4:**
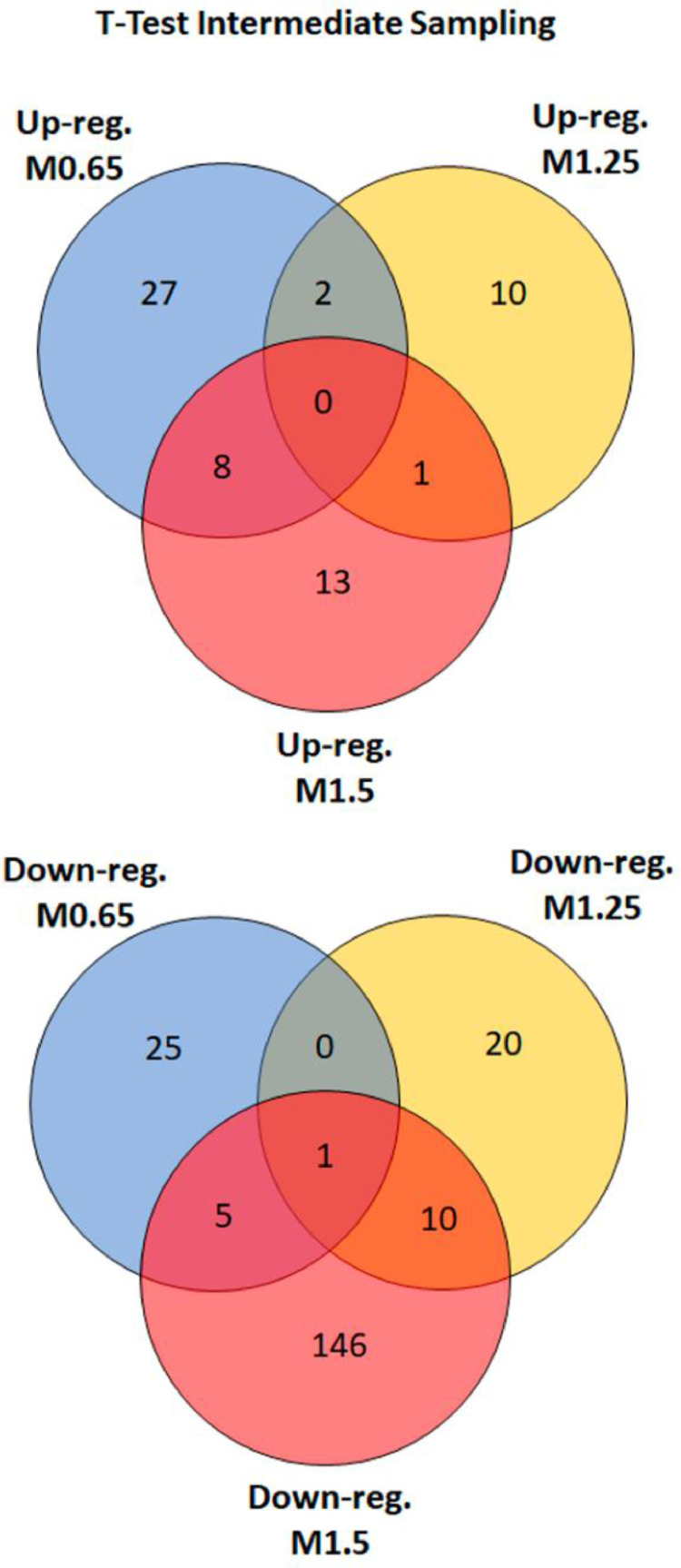
Analysis of DAP (Student's *t*-test, *p* < 0.05) between treatment versus reference diet i.e., at lower (0.77% Met-w/w) and higher (1.36% Met-w/w and 1.66% Met-w/w) concentrations in respect to the reference diet (1% Met-w/w). Venn diagrams depict the number of up-and down-regulated proteins between treatments at the 18-days experimental trial.

**Fig. 5:**
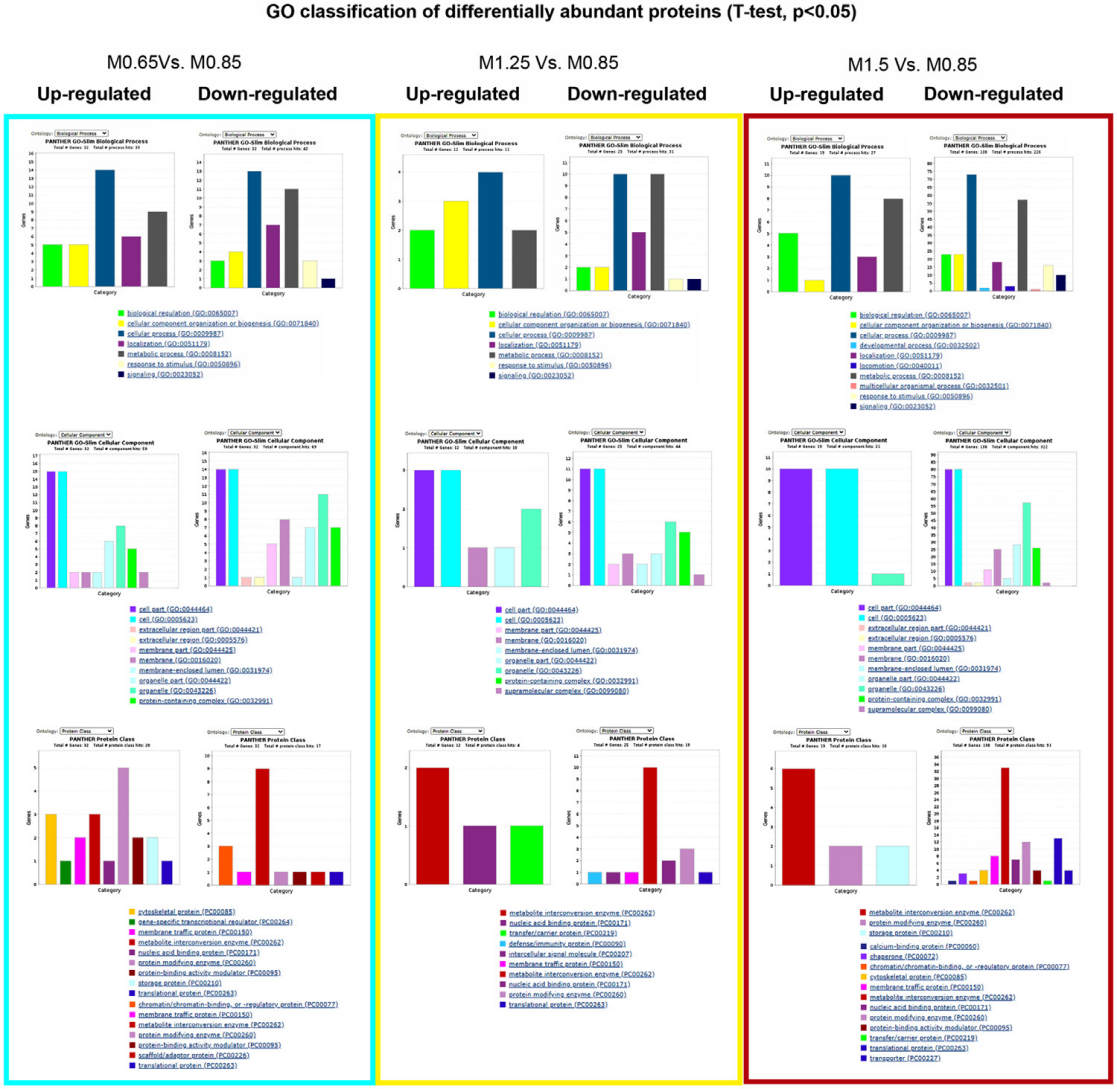
Gene Ontology (GO) classification of proteins with significant differences in abundance (Student's *t*-test, *p* < 0.05) between fish fed with Met-treatment versus reference diet (M0.85), according to Biological Process, Cellular Component and Protein class categories.

**Fig. 6:**
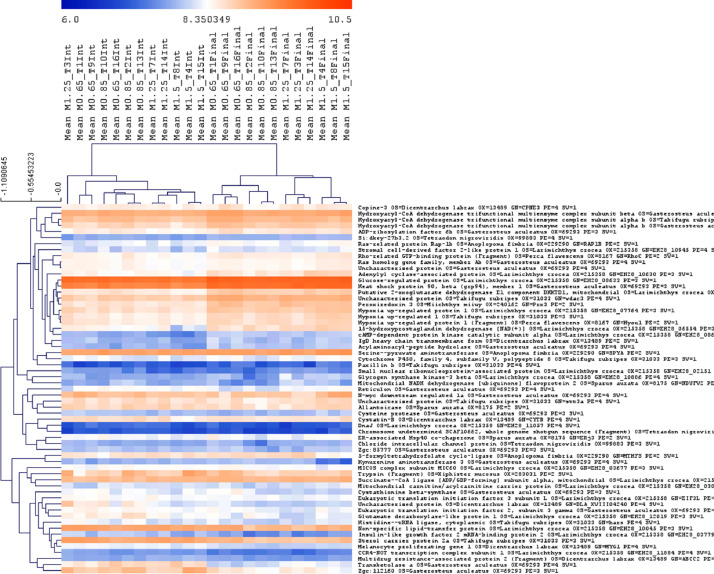
Comparison of protein pattern profiles across fish fed with diets supplemented with four different Met-concentrations at the beginning and end of the trial i.e., after 18 and 85 days. Proteins with differences in abundance (Two-way ANOVA, *p* < 0.05) and significant interaction between treatment and sampling time are shown. Mean values were calculated based on 3 liver samples/tank and triplicate tanks per treatment are indicated.

**Fig. 7:**
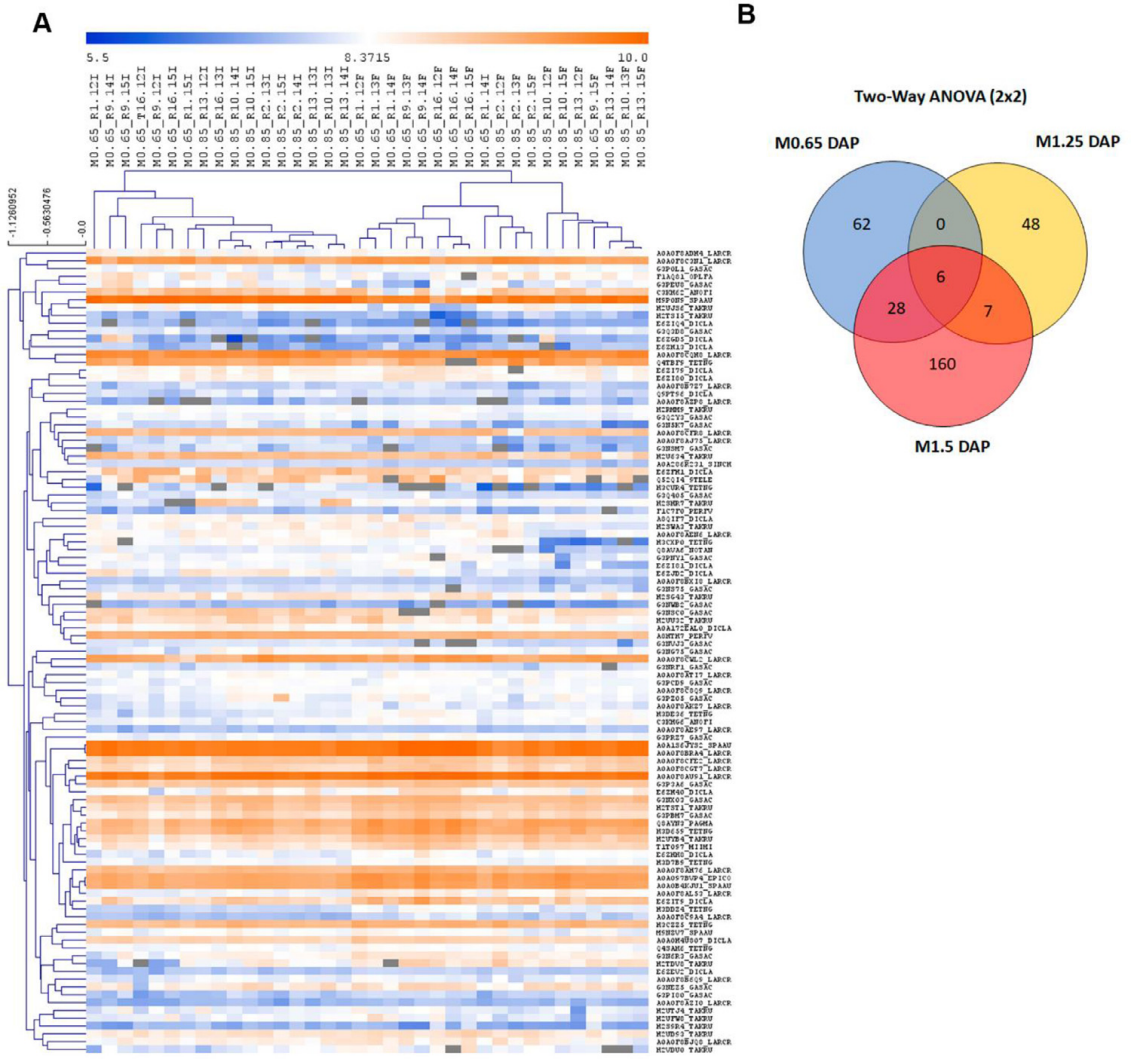
Proteins with significant differential abundance (Two-way ANOVA, *p* < 0.05) between fish fed with experimental diets in respect to the reference diet (M0.85), across two sampling time points i.e., 18 and 85 days of feeding. A) an example is provided for fish fed with M0.65 diet compared with M.0.85. B) Venn diagrams indicate the number of DAP in all pairwise comparisons between fish fed with the distinct experimental diets, across the two sampling time points.

**Fig. 8:**
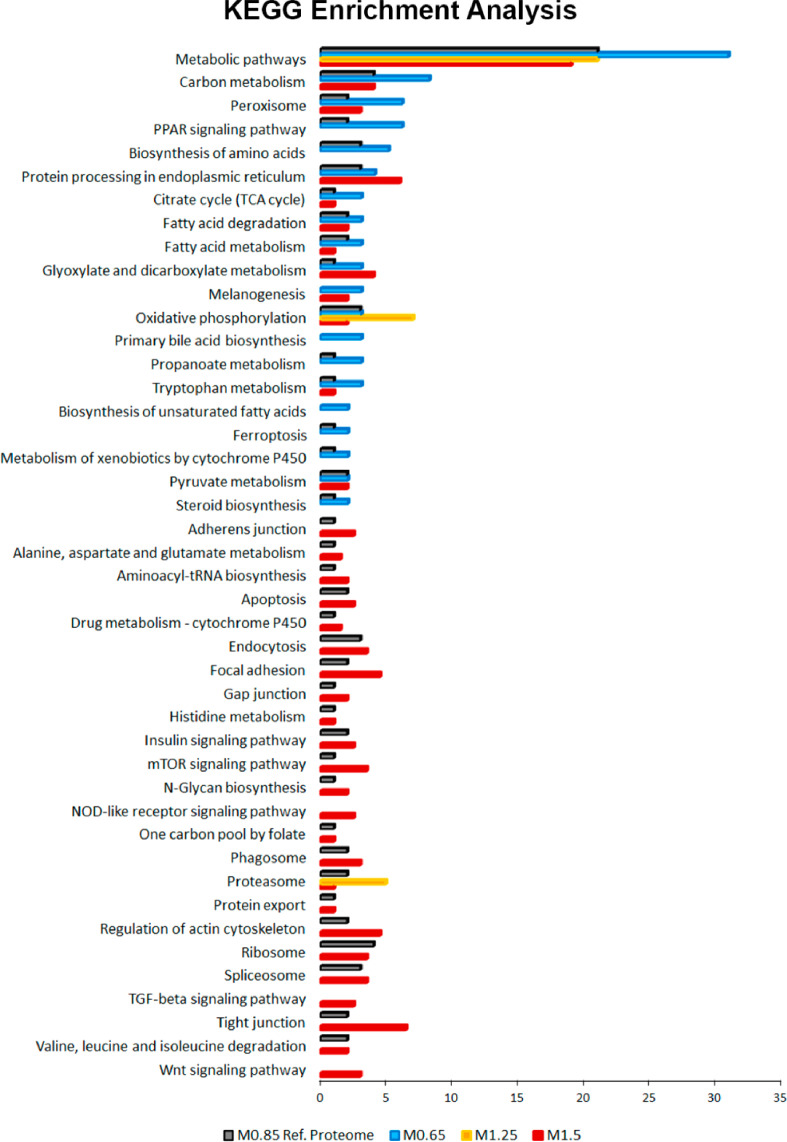
KEGG enrichment analysis (FDR, *q* < 0.05) of DAP with significant differences over time (Two-way ANOVA, *p* < 0.05) between fish fed with Met-experimental diets compared with M0.85, the reference diet.

**Fig. 9:**
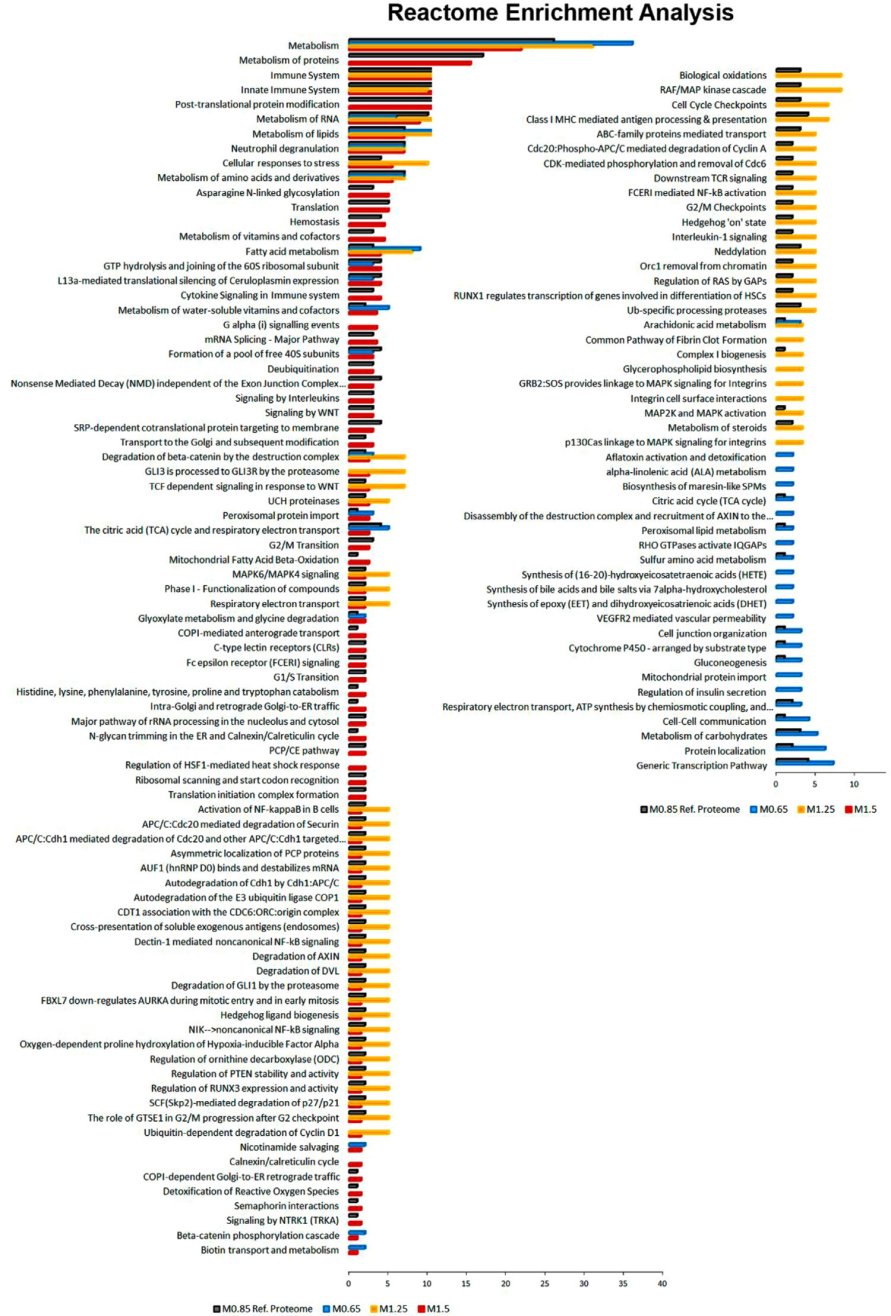
Reactome enrichment analysis (FDR, *q* < 0.05) of DAP with significant changes over time (Two-way ANOVA, *p* < 0.05) between fish fed with Met-experimental diets compared with M0.85, the reference diet.

Pairwise comparisons (Student's *t*-test, *p* < 0.05) between fish fed at 0.77% Met (w/w) (M.65), 1.36% Met (w/w) (M1.25) and 1.66% Met (w/w) (M1.5) were performed in respect to the reference/control diet (1% Met-w/w) (M0.85) formulated to be within the estimated requirement for this species i.e., 8.5 mg/g DW [3]. The major aim was to determine differential abundant proteins (DAP) responsive to specific dietary Met-levels, after 18 days of feeding. The DAP, either up-regulated or down-regulated (Fig. 4) were classified according to GO categories of BP, Cellular Component (CC) and Protein class (PC) (Fig. 5), to elucidate about their biological functions. Further comparison analyses between fish fed with the four experimental diets were established over time (after 18 and 85 days of feeding) by combining a Two-Way ANOVA (2 factors x 4 levels; *p* < 0.05) with HCL (Fig. 6). This data offered a global overview of fish samples´s relationship based on proteome fingerprinting, besides revealing proteins with significant differences after short-term and long-term feeding periods. A more detailed analysis over time was conducted between each experimental diet in respect to the reference diet (M0.85) (Two-way ANOVA, 2 factors x 2 levels; *p* < 0.05). An example is provided in Fig. 7 for M0.65 Vs. M0.85, and the total number of DAP obtained in all pairwise comparisons is shown in the Venn's diagrams. The pathways involved in the hepatic response to dietary Met-obtained by KEGG (Kyoto Encyclopedia of Genes and Genomes) metabolic and Reactome enrichment analyses (FDR, *q* < 0.05) are presented in Fig. 8, Fig. 9, correspondingly. The complete list of proteins identified by LC-MS/MS in the two feeding trials, together with the DAP analysed through uni- and multivariate statistics can be found in Supplementary Material (Appendix A).

## 2 Experimental Design, Materials and Methods

### 2.1 Fish sampling and protein sample preparation

Three European seabass juveniles were randomly selected from triplicate tanks per treatment, for proteomic analyses. Fish sampling was performed after 18 and 85 days of feeding fish with experimental diets. Before sampling, fish went through a 24 h fasting period and were lethally anaesthetized with 200 mg/L of MS-222 (Sigma Aldrich) at sampling. Nine liver samples per treatment were isolated, immediately snap-frozen in liquid nitrogen and stored at −80 °C until further analysis. Total proteins from 100 mg of liver tissue were solubilized in 7 M Urea, 2 M Thiourea, 4% CHAPS, 30 mM Tris–HCl pH 8, containing 1 mM EDTA and 1% (v/v) Protease Inhibitor Cocktail (Sigma). Liver tissues were homogenized using an Ultra-Turrax IKA T8 (IKA-WERG), followed by sonication (5 cycles of 5 s each) and two centrifugation steps at 13,000 × g for 10 min, at 4 °C. The final supernatants were cleared from contaminants using a ReadyPrep™ 2-D Cleanup kit (Bio-Rad) and total proteins were quantified using the Quick Start™ Bradford Protein Assay using BSA as standard (Bio-Rad). The cleaned protein pellet was further resuspended in 100 mM Tris pH 8.5, 1% sodium deoxycholate, 10 mM tris (2-carboxyethyl)phosphine (TCEP), 40 mM chloroacetamide and protease inhibitors for 10 min at 95 °C at 1000 rpm (Thermomixer, Eppendorf). Peptide samples were prepared following the solid-phase-enhanced sample-preparation (SP3) protocol as described in [4]. Enzymatic digestion was performed with 2 µg Trypsin/LysC overnight at 37 °C at 1000 rpm.

### 2.2 Liquid chromatography coupled to tandem mass spectrometry (LC-MS/MS)

Peptides were analysed through reverse phase nano liquid chromatography by using an Ultimate 3000 system coupled online to a Q-Exactive Hybrid Quadrupole-Orbitrap mass spectrometer (Thermo Scientific, Bremen, Germany). Samples were loaded onto a trapping cartridge (Acclaim PepMap C18 100Å, 5 mm x 300 µm i.d., 160,454, Thermo Scientific) in a mobile phase of 2% ACN, 0.1% FA at 10 µL/min. After 3 min loading, the trap column was switched in-line to a 50 cm by 75 μm inner diameter EASY-Spray column (ES803, PepMap RSLC, C18, 2 μm, Thermo Scientific, Bremen, Germany) at 300 nL/min. Separation was generated by mixing A: 0.1% FA, and B: 80% ACN, with the following gradient: 5 min (2.5% B to 10% B), 120 min (10% B to 30% B), 20 min (30% B to 50% B), 5 min (50% B to 99% B) and 10 min (hold 99% B). Subsequently, the column was equilibrated with 2.5% B for 17 min. Data acquisition was controlled by Xcalibur 4.0 and Tune 2.9 software (Thermo Scientific, Bremen, Germany).

The mass spectrometer was operated in data-dependent acquisition (DDA) positive mode alternating between a full scan (*m/z* 380–1580) and subsequent HCD MS/MS of the 10 most intense peaks from full scan (normalized collision energy of 27%). ESI spray voltage was 1.9 kV. Global settings: use lock masses best (*m/z* 445.12003), lock mass injection Full MS, chrom. peak width (FWHM) 15 s. Full scan settings: 70k resolution (*m/z* 200), AGC target 3e6, maximum injection time 120 ms. DDA settings: minimum AGC target 8e3, intensity threshold 7.3e4, charge exclusion: unassigned, 1, 8, >8, peptide match preferred, exclude isotopes on, dynamic exclusion 45 s. MS2 settings: microscans 1, resolution 35k (*m/z* 200), AGC target 2e5, maximum injection time 110 ms, isolation window 2.0 *m/z*, isolation offset 0.0 *m/z*, spectrum data type profile.

### 2.3 Protein identification

#### 2.3.1 Database searching

MS/MS samples were analysed using Sequest (Thermo Fisher Scientific, San Jose, CA, USA; version IseNode in Proteome Discoverer 2.2.0.388) and searched against the UniProt database for the *Eupercaria* taxonomic selection (Release 10_2018; 194,001 entries). The Sequest HT search engine was used to identify tryptic peptides. The ion mass tolerance was 10 ppm for precursor ions and 0.02 Da for fragmented ions. The maximum of allowed missing cleavage sites was two. Cysteine carbamidomethylation was defined as constant modification. Methionine oxidation and protein N-terminus acetylation were defined as variable modifications. Peptide confidence was set to high and only those with at least six amino acids were considered for identification. The processing node Percolator was enabled with the following settings: maximum delta Cn 0.05; decoy database search target FDR 1%, validation based on q-value. Protein label free quantitation was performed with the Minora feature detector node at the processing step. Precursor ions’ quantification was performed at the processing step with the following parameters: peptides to use unique plus razor, precursor abundance was based on intensity, normalization mode was based on total peptide amount, pairwise protein ratio calculation, hypothesis test was based on ANOVA (background based). The Magellan storage files (MSF) were imported into Scaffold (version Scaffold_4.8.9, Proteome Software Inc., Portland, OR) to validate MS/MS based peptide and protein identifications, and perform quantitative analysis based on extracted ion chromatograms and for the TOP3 resulting most intense ion peak areas. A second database search was conducted by using the X! Tandem algorithm (The GPM, thegpm.org; version X! Tandem Alanine 2017.2.1.4 to increase/improve protein identification. X!Tandem was set up to search also for a reverse concatenated UniProt_Eupercaria_2018_10 database (421,118 entries), besides UniProt_Eupercaria_2018_10 database. Trypsin digestion was assumed, together with a parent ion tolerance of 10 ppm and fragment ion mass tolerance of 0.020 Da. Carbamidomethyl of cysteine was specified as a fixed modification, and Glu->pyro-Glu-of the N-terminus, ammonia-loss of the N-terminus, gln->pyro-Glu-of the N-terminus, oxidation of methionine and acetyl of the N-terminus were specified in X! Tandem as variable modifications

#### 2.3.2 Criteria for protein identification

Peptide identifications in the Scaffold software (version Scaffold_4.8.9, Proteome Software Inc., Portland, OR) were accepted if they could be established with a probability greater than 95% by the Peptide Prophet algorithm [5] with Scaffold delta-mass correction. Protein identifications were accepted if they could be established at greater than 99% probability, Decoy FDR < 0.1%. and contained at least 2 identified peptides (peptide Decoy FDR < 0.01%). Protein probabilities were assigned by the Protein Prophet algorithm [6]. Proteins that contained similar peptides and could not be differentiated based on MS/MS analysis alone, were grouped to satisfy the principles of parsimony. The mass spectrometry data have been deposited on ProteomeXchange Consortium via the PRIDE partner repository with the data identifiers PXD019610 and 10.6019/PXD019610.

## 3 Data Analysis

Scaffold output files were merged for comparison between the 4 sets of experimental diets consisting of 9 MS/MS runs per treatment (3 fish x 3 tanks), in a total of 36 samples. A total of 2610 proteins present in at least 2/3 of all replicates (in each treatment) across the 2 feeding trials (Appendix A), were considered as reproducible for further statistical analysis. Protein abundance was estimated based on TOP3 Precursor Intensity and normalized values (against the sum of all ion intensities in each sample replicate) were used for further statistical analysis. The precursor intensity was selected for quantitation of protein relative abundance because it offers a good compromise between the accuracy of labelled techniques and the simplicity and lower cost of label-free methods. It relies on the measurement of the signal intensity of the peptide precursors representing a specific protein at the MS level and comparison of these intensities across samples. The TOP3 precursor intensities were log10 transformed prior to any statistical analysis to ensure a Gaussian distribution of the residuals. The residuals’ normality and homoscedasticity across treatments was confirmed on the IBM™ SPSS™ Statistics software V25.0.

Normalized and log transformed proteomics data were imported into the MultiExperiment Viewer software [7] version 4.9 to combine one-way ANOVA (*p* < 0.05) or Student's *t-*test (*p* < 0.05) with hierarchical clustering analysis (HCL). The average linkage method was selected for clustering and the Pearson correlation (similarity distances) was taken as the metric distance. Proteins with most significant differences across all samples determined by one-way ANOVA (*p* ≤ 0.01) were used for Principal Component Analysis (PCA) using the MeV software.

GO classification of proteins with significant differences (One-Way ANOVA, *p* < 0.05) across treatments, according to Biological Process (BP), was performed with the PANTHER 14.1 classification system [8] generated from the 2018_04 release of Reference Proteome dataset. Proteins with significant differences between each treatment in respect to the reference/control diet (Student's *t*-test, *p* < 0.05) were further analysed by Gene Ontology (GO) and pathway enrichment analysis as described in the following section. Venn's diagrams to elucidate about the number of common and unique proteins between experimental diets were obtained with Venny 2.1 [9].

### 3.1 Pathway enrichment analysis

FASTA protein sequences were blasted on STRING (Search Tool for the Retrieval of Interacting Genes/Proteins) database v.11 (Szklarczyk et al., 2019) to search for Danio rerio homologs. The matched proteins on STRING database with an identity of at least 60% and high bit score, were selected for KEGG and Reactome pathway enrichment analyses (FDR, *q* < 0.05). An internal “reference” seabass liver proteome based on fish fed with M0.85 diet (fullfilling the species Met-requirements) was settled to determine which proteins could be over-represented in control conditions in respect to the proteome in *Danio rerio*. After searching in the STRING database, 1721 unique entries were sucessfully matched as *D. rerio* homologs to establish the European seabass reference proteome.

## Ethics Statement

All experiments complied with ARRIVE guidelines and were carried out in accordance with European laws (2010/63/EU) and Portuguese legislation for the use of laboratory animals (DL n°113/2013, 7 August). This study was approved by the ORBEA Animal Welfare Committee of CCMAR and the Food and Veterinary Medicine Directorate (DGAV), the Portuguese competent authority for animal welfare and protection, under license number 014,781.

## Declaration of Competing Interest

The authors declare that they have no known competing financial interests or personal relationships which have, or could be perceived to have, influenced the work reported in this article.

## Supplementary Material

**Appendix A**. Supplementary data is presented in Excel file containing distinct spreadsheets corresponding to the list of proteins commonly identified in the liver across the two feeding trials, and to DAP with significant differences analysed by different statistical methods. Protein annotation according to STRING satabase is also presented.

## Appendix B Supplementary materials

Image, application 1
